# Diagnostic accuracy of Lipoarabinomannan detection by lateral flow assay in pleural tuberculosis

**DOI:** 10.1186/s12879-024-09088-4

**Published:** 2024-02-09

**Authors:** Atish Mohapatra, Ujjwala Gaikwad, Ranganath T. Ganga, Pratibha Sharma

**Affiliations:** 1 Department of Microbiology, All India Institute of Medical Sciences Raipur, Tatibandh, G E Road, Raipur, 492099 India; 2 Department of Pulmonary Medicine, All India Institute of Medical Sciences, Raipur Tatibandh, G E Road, Raipur, 492099 India; 3 Department of Microbiology, Shri Balaji Institute of Medical Sciences, Mowa, Raipur, Chhattisgarh, 492001 India

**Keywords:** Lipoarabinomannan, Pleural tuberculosis, Adenosine deaminase

## Abstract

**Background:**

Lipoarabinomannan (LAM) antigen serves as an attractive biomarker to diagnose Tuberculosis (TB). Given the limitations of current diagnostic modalities for Pleural TB, current study evaluated LAM’s potential to serve as a point-of-care test to diagnose pleural TB.

**Methods:**

A cross sectional, diagnostic accuracy study was conducted during February to November 2021 in a tertiary care hospital in India. LAM antigen detection was performed on pleural fluid as well as early morning urine specimen of suspected pleural TB patients by “Alere/ Abott Determine TB LAM” lateral flow assay (LAM-LFA). The results were compared to microbiological reference standards/MRS (Mycobacterial culture or NAAT) and Composite reference standards/CRS (MRS plus Clinico-radiological diagnosis).

**Results:**

A total of 170 subjects were included in the analysis, including 26 with Definite TB, 22 with Probable TB, and 122 with No TB. Compared to MRS and CRS, the sensitivity (61.54% & 45.83%) and positive predictive value (PPV) (57.14 & 78.57%) of Pleural LAM-LFA testing were found to be suboptimal, whereas the specificity (91.67% & 95.08%) and negative predictive value (NPV) (92.96% & 81.69%) of the assay were found to be good. Urinary LAM-LFA performed even worse than pleural LAM-LFA, except for its higher specificity against MRS and CRS (97.2% and 98.3%, respectively). Specificity and PPV of pleural LAM detection increased to 100% when analysed in a subgroup of patients with elevated ADA levels (receiver operating curve analysis-derived cut off value > 40 IU/ml).

**Conclusion:**

Detection of LAM antigen by LFA directly from pleural fluid was found to be a useful test to predict absence of the disease if the test is negative rather than using as a POCT for diagnosis.

## Introduction

Pleural tuberculosis (PTB) diagnosis remains an unsolved problem worldwide due to its paucibacillary nature, which results in suboptimal performance of current tools for its laboratory detection. Despite being a WHO-recommended diagnostic tool, GeneXpert’s poor efficacy when evaluating pleural effusion specimens makes it less useful in suspected pleural TB patients. The gold standard for pleural TB diagnosis is the detection of *M. tuberculosis* in pleural fluid or pleural biopsy by culture or histopathological examination of caseating granulomas in the pleura, combined with the detection of acid-fast bacilli [1]. However, the need for a trained medical professional and invasive nature of the procedure makes it less feasible. The sensitivity of pleural fluid culture is suboptimal when used alone or in combination with other biomarkers and molecular methods.

Due to its excellent correlation with disease status and feasibility in routine laboratory settings, measurement of pleural Adenosine deaminase (ADA) levels has traditionally provided significant aid in diagnosing clinicoradiologically directed pleural TB [2]. Similarly other unexplored biomarkers with the potential for diagnosis are being investigated to see if they can meet the WHO criteria for biomarkers-based tests for extrapulmonary TB diagnosis. According to WHO, biomarkers-based non-sputum test should have high specificity (up to 98%) and sensitivity (up to 80%) against Microbiological Reference Standards (MRS) to serve as an adjunct to current microbiological diagnostic modalities for tuberculosis [3].

Lipoarabinomannan (LAM), a mycobacterial cell wall antigen which is secreted in urine and other sterile body fluids [4], has been evaluated as a promising biomarker for the diagnosis of tuberculosis. In 2015, the WHO recommended the TB LAM test in lateral flow format as a low-cost, rapid bedside test for diagnosing tuberculosis in patients who are HIV positive with a CD4 count of 100 cells/L or in severely ill HIV patients. Since there is evidence that LAM is secreted in sterile body fluids, many researchers investigated its direct detection in non-urine specimens such as CSF, pericardial fluid, and pleural fluid, with promising results [4–7].

However, its accuracy in pleural TB diagnosis was demonstrated in only a few studies, which reported suboptimal sensitivity but excellent specificity [7, 8]. Its detection in a lateral flow assay format is the most convenient option for use as a point-of-care test. Given its promising results and the limitations of existing diagnostic tools, it is necessary to investigate the diagnostic potential of this biomarker further. Moreover, no such work on pleural TB diagnosis has yet been reported in Indian literature. Given the above, the current study was designed to assess the diagnostic accuracy of TB-LAM antigen detection by lateral flow assay format from pleural fluid and urine specimens of the patients with suspected and/ confirmed pleural TB against microbiological and composite reference standards.

## Materials and methods

### Study design and participants

A prospective diagnostic accuracy study was conducted in the departments of Microbiology & Pulmonary Medicine at All India Institute of Medical Sciences, Raipur, Chhattisgarh, which is a tertiary care institute of national importance under Ministry of health and family welfare, Government of India. In a cross-sectional design, the study was conducted for ten months (February– November 2021). Study participants included patients aged > 18 years with signs and symptoms of tubercular pleural effusion for whom testing by both liquid Mycobacterial culture and Nucleic acid amplification test (NAAT) were requested by the clinician as a part of standard treatment care. Such patients were traced back from the laboratory to the pulmonary ward/OPD to obtain the clinical details were consecutively recruited after taking informed consent. Patients on antitubercular treatment for present illness for > 2 months, had completed the full course of antitubercular treatment in the past, those whose specimen grew Non-Tuberculous Mycobacteria on culture or patients not traceable for full work up were excluded from the study. The study was reported in accordance with the STARD guidelines.

### Study procedures

The investigator used a standard proforma to collect demographic and clinical information about the illness as well as biochemical profiles of pleural fluid, including ADA, LDH, sugar, and proteins. LAM testing was done on pleural fluid samples from patients that had been sent to the laboratory for Mycobacterial culture/CBNAAT/both (index test). After processing for mycobacterial culture using BD BACTEC MGIT 960 system (BD life sciences, Becton, Dickinson U.K. Limited) and NAAT testing using either Xpert/MTB RIF system (Cepheid, Sunnyvale, USA) or TrueNat (Molbio diagnostics, Goa, India), the samples were stored in the refrigerator until LAM testing. Due to Xpert/MTB-RIF cartridge shortages in India during study period, most samples were tested with TrueNat (July– November 2021). On the following day, an early morning urine sample was collected from the same patients and tested for TB-LAM.

#### LAM lateral flow assay (LFA)

The assay was performed on remaining pleural fluid and early morning urine without centrifugation. 60 µL of each pleural fluid and urine specimen was added to the “Determine TB LAM” lateral flow assay test strip (Make– Alere/Abott; USA). After 25 min, the test strip showed coloured bands at “Test” and “Control,” indicating a positive result. Two observers independently read the test strip by comparing the result to the manufacturer-supplied reference card (showing intensity grading from + 1 to + 4) and the test positivity was determined by grade + 1. Interpreters of the LAM-LFA test were blinded to patient clinical data and tuberculosis diagnosis by other methods. Any disagreements between interpreters were resolved by a third interpreter. Observers of reference tests were blinded to index test results.

Because pleural fluid contains proteins that may mask the free presence of LAM (Matrix phenomenon) in the sample, the pleural fluid samples were pretreated to mitigate the effect. All pleural fluid specimens positive for MGIT/NAAT or both but negative for LAM-LFA were pretreated using the perchloric acid treatment method proposed by Laurentius et al. [9]. The pretreated pleural fluid was subjected to the TB LAM antigen assay again, and the results were recorded.

#### Clinical severity assessment

The clinical severity of pleural tuberculosis was assessed using modified Bandim scoring (MBS) by assessing five symptoms (Cough, Hemoptysis, Dyspnea, Chest pain, Night sweats) and five signs (Anemia, Pulse > 90 beats/min, Abnormal lung auscultation, Temperature > 37 °C, Mid Upper Arm Circumference (MUAC) < 220 mm) since BMI data was unavailable [10]. Symptoms/signs were scored “1” if present and “0” if absent. The total scores were used to classify patients as mild (score 0–5), moderate (6–7), or severe (score 8) disease. Severity was correlated with pleural fluid LAM positivity grade. Pleural tuberculosis illness duration was divided into two categories: >1 week and < 1 week [11].

#### Reference standards for comparison

Results of LAM testing was compared against Microbiological reference standards (MRS) that comprised of patients with “Definite TB” and Composite Reference standards (CRS) comprising of “Definite TB” as well as “Probable TB” patients. “Definite TB” included patients with clinico-radiological picture consistent with pleural TB along with positive MTB results by either of Culture/CBNAAT/both done on the pleural fluid. “Probable TB” cases included patients with clinical/radiological features of pleural TB that tested negative by both Culture/CBNAAT and were treated for TB with demonstrable treatment response within 3–4 months. “Non -TB” patients were those with negative microbiological tests results and for whom alternative diagnosis was made on histological or radiological findings and not treated for TB.

#### Data analysis

The data was analyzed using SPSS Ver 22·0 software for all parameters. Performance of LAM assay was determined by measuring its Sensitivity, specificity, PPV and NPV against both the reference standards and were reported with Wilson 95% confidence intervals (95% CI). The statistical significance was calculated by using Chi-square test. *p* value of < 0·05 was taken as measure of statistical significance. Concordance between tests were measured using the kappa co-efficient. Receiver Operating Characteristic (ROC) curve was plotted to find out optimum cut off values for LDH and ADA in pleural fluid at which diagnosis can be made when compared to MRS and CRS. The correlation between parameters were analyzed by Spearman’s test.

## Results

**Fig. 1:**
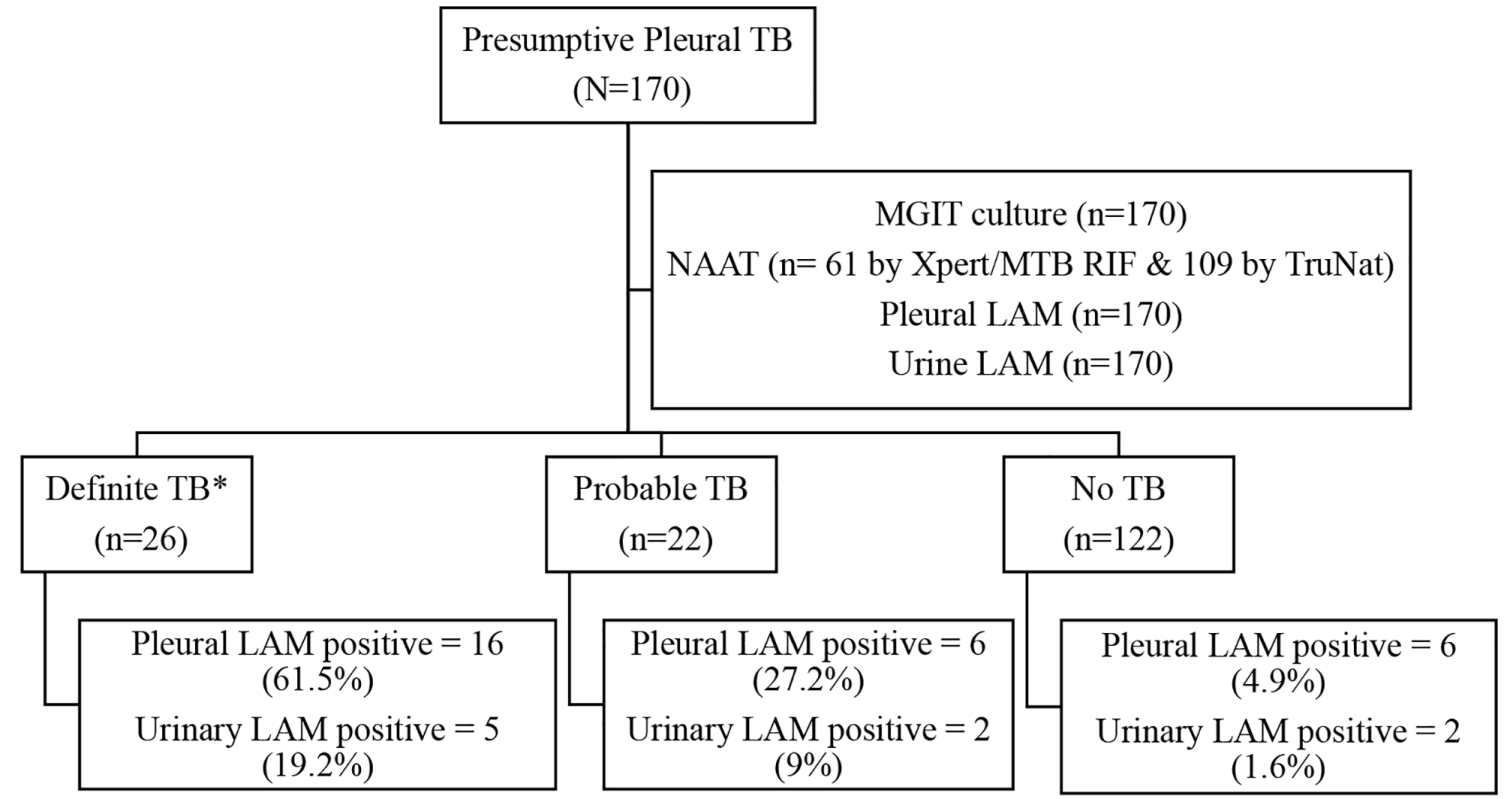
Study Flow chart depicting patient’s recruitment and sampling workflow *Note - Out of the 26 ‘Definite TB’, seven were diagnosed only on the basis of NAAT, eight were diagnosed only on the basis of culture by MGIT whereas eleven were diagnosed on the basis of both MGIT culture & NAAT

A total of 170 presumptive pleural TB patients were enrolled in the study and categorized into DTB (Definite TB), PTB (Probable TB) and NTB (No-TB) (Fig. 1). The demographic and clinical details of the patients is described in Table 1. Maximum number of patients belonged to young age group with the mean age of 49·21 (SD ± 17·4) and males outnumbered females with male: female ratio of 2·26:1.

**Table 1:** Demographic and Clinical details of the study population

	DTB	PTB	NTB	All patients	*P* value
**n**	26	22	122	170	
**Age**					
Young (18–47 years)	16 (33·3)	13 (27·0)	45 (36·8)	74 (43·5)	0·008
Middle (48–63 years)	8 (16·6)	3 (6·2)	46 (37·7)	57 (33·5)	0·09
Elderly (> 64 years)	2 (4·1)	6 (12·5)	31 (25·4)	39 (22·9)	0·3
**Gender**					
Male	18(69·2)	16 (72·7)	84 (68·8)	118 (69·4)	0·9
Female	8 (30·7)	6(2·0.3)	38 (31·1)	52 (30·5)	
**Clinical details**					
Average duration of illness in weeks (± SD)	4·11 ± 2·45	3·59 ± 2·50	3·53 ± 2·17	3·62 ± 2·25	0·31
Fever	21 (80·7)	16 (7·0.7)	69 (56·5)	106 (62·3)	0·01
Cough	24 (92·3)	18 (81·8)	99 (81·1)	141 (82·9)	0·32
Chest pain	20 (76·9)	12 (54·5)	76 (62·2)	108 (63·5)	0·38
Dyspnea	23 (88·4)	19 (86·3)	113 (92·6)	155 (91·1)	0·28
Weight loss*	21 (80·7)	10 (45·4)	57 (46·7)	88(51·7)	0·03
**Site of pleural effusion on radiological examination**					
Unilateral	20 (41·6)	15 (31·2)	72 (59·0)	159 (93·5)	0·09
Bilateral	6 (12·5)	7 (14·5)	50 (40·9)	63 (37·0)	
**Associated Risk factors**					
HIV	3 (11·5)	2 (9·0)	0 (0·0)	5 (2·9)	0·001
Malnutrition (**MUAC < 22 cm)	6 (23·0)	3 (13·6)	1 (0·8)	10 (5·8)	0·00001
Malignancy	2 (7·6)	0	7 (5·7)	9 (5·2)	1·00
Chronic Kidney Disease	2 (7·6)	0	1 (0·8)	3 (1·7)	0·19
Chronic Liver Disease	0	1 (4·5)	2 (1·6)	3 (1·7)	1·00
Chronic Heart Disease	0	0	4 (3·2)	4 (2·3)	0·57
COVID-19	5 (19·2)	1 (4·5)	25 (20·4)	31 (18·2)	0·27
Diabetes Mellitus	1 (3·8)	4 (18·1)	33 (27·0)	38 (22·3)	0·02
Hypertension	6 (23·0)	2 (9·0)	39 (31·9)	47 (27·6)	0·05
Smoking	11 (42·3)	9 (40·9)	39 (31·9)	59 (34·7)	0·28
Associated Pulmonary TB	7(26·9)	1(4·5)	0 (0·0)	08 (4·7)	0·001
**Biochemical analysis of Pleural fluid** ^**#**^					
Protein (g/DL)	4·6 (3·1–5·7)	4·7 (4·0–5·3)	4·3(2·5–5·4)	4·4(2·7 − 5·5)	0·158
Glucose (mg/dl)	44·5 (10·0–73·25)	62·0 (10·0-114·7)	82·0 (51·5-114·0)	77 (40–113·5)	0·009
LDH (IU/L)	575·0 (228·0-1081·0)	551·5 (294·7-728·2)	325·5 (154·5-651·5)	370 (175·5-748·5)	0·023
ADA (IU/L)	59·4 (42·9–88·2)	63·0 (51·4–92·9)	13·68 (10·0–22·7)	18 (10·0–44·4)	< 0·006

Data presented as n (%) unless other wise stated

**≥2 kg or > 5% initial body weight

*Mid upper arm circumference (MUAC) was recorded using a plastic measuring tape. The right upper arm was measured at the midpoint between the tip of the shoulder and the tip of the elbow (olecranon process and the acromion process)

^#^Estimates of levels of biochemical parameters tested are presented as median (Interquartile range)

The study population had smoking as the most common risk factor in all groups. Upon clustering the two groups (DTB & PTB) as TB, we found that there was significant intergroup difference between TB and NTB cases for HIV and malnutrition as risk factors. About 5/48 (10·41%) of TB patients were HIV positive whereas none of the patients in NTB group were HIV positive (*p* = 0·0001). Nine out of 48 (18.75%) of TB patients had malnutrition versus only one out of 122 (0·8%) Non-TB patients (*p* = 0·00001).

**Fig. 2:**
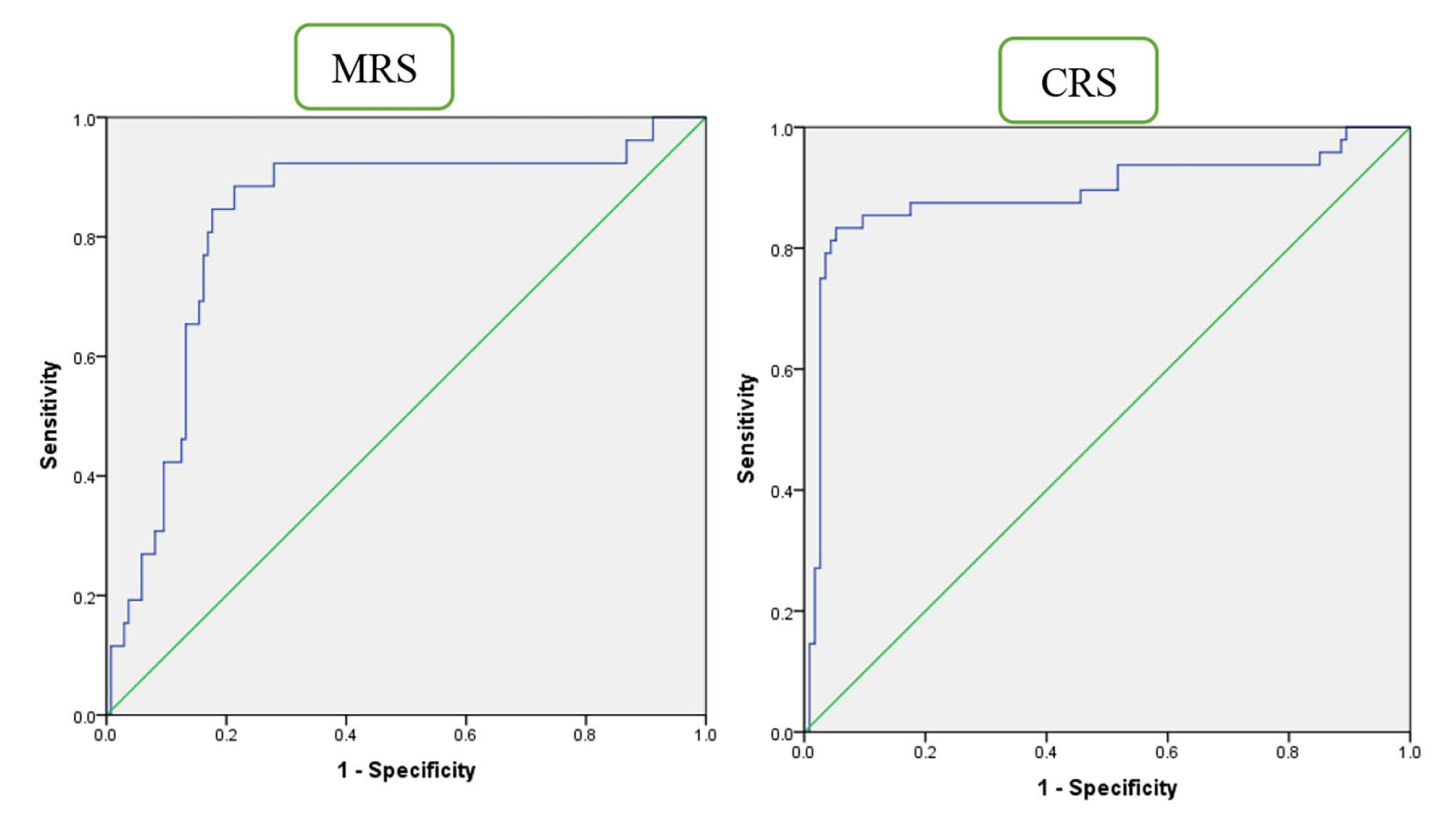
ROC analysis of ADA levels in pleural TB against MRS& CRS

Among the biochemical parameters, LDH and ADA were assessed to have strong differentiating ability between TB and Non-TB cases (*p* = 0·023 and *p* < 0·006 respectively). To find out the exact cut off value at which presence of disease can be predicted in microbiologically confirmed and clinically diagnosed disease, an ROC analysis was performed taking MRS and CRS as standards for comparison. When compared to MRS and CRS, the sensitivity and specificity of LDH was the highest at an Area under the curve (AUC)-derived cut-point of 364·5 IU/L and 442·0 IU/L respectively, and the AUC was 0·619 and 0·645 respectively suggesting it to be a “poor” biomarker. While, the sensitivity and specificity of ADA was the best at an AUC derived cut off point of 31·5 IU/L and 40·0 IU/L respectively against MRS and CRS and the area under the ROC curve was 0·829 and 0·889 respectively (Fig. 2).

### Results of LAM testing on pleural fluid and urine

Overall, LAM antigen positivity in pleural fluid was 16·4% (28/170), while urinary LAM positivity was observed to be just 5·2% (9/170). Hence, performance of TB LAM antigen detection was significantly better (*p* = 0·006) when tested directly from pleural fluid over urine of the same patient group. This finding was consistent in all three categories of the patients with highest positivity (61·5%) found in the pleural fluid of patients with definite TB. There were false positive pleural LAM results in 6/122 (4·9%) NTB cases, out of which two patients (1·6%) also showed false positive results on urinary LAM. The overall concordance between pleural LAM and Urinary LAM antigen in detecting TB was 85·3% (145/170) with “fair” inter-test agreement. (cohen’s kappa = 0·265).

LAM LFA demonstrates positivity in grades ranging from 1 + to 4+, which is proportional to the amount of antigen present in the sample. When tested for pleural fluid, positivity was seen in all ranges, i.e., from 1 + to 4+, as opposed to urine, where only 1 + and 2 + were obtained. However, among all the samples tested, grade 1 + was the most prominently observed, accounting for 26·9%, 18·1%, and 2·4% of DTB, PTB, and NTB cases in pleural LAM and 11·5%, 9·0%, and 1·6% of DTB, PTB, and NTB cases in urinary LAM.

Pretreatment with perchloric acid did not affect LAM positivity in negative samples. Ten of twenty-six samples (38·4%) were pretreated with perchloric acid and tested for LAM, but none were positive.

### Diagnostic performance of Pleural and urinary LAM against reference standards

**Fig. 3:**
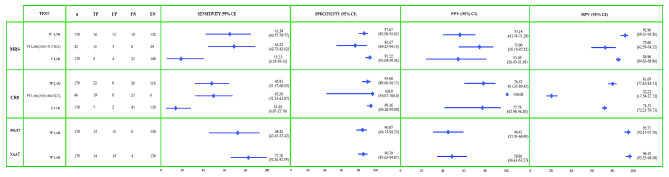
Diagnostic accuracy of pleural and urine TB LAM Antigen detection against various standards for comparisonFootnotes for Fig. 3:PF LAM = Pleural fluid Lipoarabinomannan antigen detection; U LAM = Urine Lipoarabinomannan antigen detectionADA = Adenosine DeaminaseCI = Confidence interval; PPV = positive predictive value; NPV = Negative predictive value;MRS = Microbiological Reference Standards (Pleural Fluid tested positive for MTB by Liquid culture/NAAT/both)CRS = Composite Reference Standards (Clinico-radiological plus Microbiologically confirmed disease)MGIT = Mycobacterial Growth Indicator Tube/Liquid culture for MtbNAAT = Nucleic Acid Amplification Test (CBNAAT/Truenat)

The Pleural TB LAM assay was able to correctly identify 61·5% (16/26) of microbiologically confirmed TB cases (definite TB/MRS), but only 45·8% (22/48) of all probable and definite TB cases (CRS). Consequently, its sensitivity to identify diseased populations was suboptimal. However, it performed well in identifying those without pleural TB, with a specificity of 91·67% against MRS and a specificity of 95·08% against CRS. Similarly, the test revealed a very low PPV against MRS (57·14%) that increased slightly against CRS (78·57%). Nevertheless, the NPV was high at 92·96 and 81·69% against MRS and CRS, respectively, making it a useful test for predicting the absence of the disease if the test is negative (Fig. 3).

Compared to the individual microbiological tests, Pleural LAM detected 13/19 (68.4%) and failed to detect 6/19 (31·6%) culture-positive samples. Similarly, it detected 14/18 (77.8%) and missed 4/18 (22·2%) NAAT-positive samples. LAM falsely identified 14/152 (9·2%) NAAT-negative samples and 15/151 (9·9%) culture-negative samples. Consequently, the overall diagnostic accuracy (Fig. 3) and concordance between LAM and NAAT (89.41%) was superior to that of LAM and Culture (87·64%) with a moderate correlation (Pearson’s co-efficient of 0·49 for culture and 0·52 for NAAT) between each of these tests.

The diagnostic performance of urinary LAM was poor when compared to pleural fluid. The LAM urine assay correctly identified only 5/26 cases against MRS and 7/48 against CRS, yielding extremely low sensitivity of 19·2% and 14·5%, respectively. However, the specificity was significantly higher than that of pleural LAM (97·22% and 98·36%, respectively). The PPV was comparable to that of pleural LAM, while the NPV was significantly lower.

In a subset analysis, the performance of LAM testing was examined in patients with significant ADA values, as previously described, to determine whether LAM testing can improve the diagnostic value of ADA in such patients. While the sensitivity was slightly increased (over the original) against both MRS and CRS, the specificity and PPV of LAM testing were dramatically increased to 100% when compared to CRS (Fig. 3).

### Association of host factors with LAM positivity

Analysis of host factors with pleural LAM positivity revealed malnutrition and severity of illness as the major factors affecting LAM positivity. Odds of getting pleural LAM positivity was 5·6 times higher in malnourished patients (*p* = 0·06). Similarly, there was a significantly increased risk of pleural LAM positivity in patients having severe disease as compared to mild and moderate disease. A strong correlation between grades of LAM positivity by either of the specimen was found with severity of illness. The correlation between grade of LAM positivity (Spearman’s rho [r_s_ (46) = 0·44]) with clinical severity was stronger in urine when compared against pleural fluid (Spearman’s rho [r_s_ (46) = 0·39]) (Table 2).

**Table 2:** Association of host factors with pleural LAM positivity in confirmed and suspected pleural TB cases (*n* = 48)

		*n* = 48	Pleural LAM positivity (%)	Odds ratio (95%CI)	*p*
Duration of illness	> 1 week	43	21 (48·8%)	3·81 (37·01 − 0·39)	0·35
	< 1 week	5	1 (20·0%)		
Disease severity	Mild	15	4 (26·0%)	0·08 (0·54 − 0·01)	0·07
	Moderate	22	9 (40·9%)	0·15 (0·88–0·02)	0·52
	Severe	11	9 (81·8%)	1·00	Ref
HIV status	Positive	5	2 (40·0%)	0·76 (5·05 − 0·11)	1·00
	Negative	43	20 (46·5%)		
Malnutrition	Yes	9	7 (77·7%)	5·6 (30·61 − 1·02)	0·06
	No	39	15 (38·4%)		
Diabetes Mellitus	Yes	5	4 (80·0%)	5·55(53·96 − 0·57)	0·16
	No	43	18 (41·8%)		
Associated Pulmonary TB	Yes	8	5 (62·5%)	2·25(10·75 − 0·47)	0·44
	No	40	17 (42·5%)		
ADA level > 40 IU/ml	Yes	40	19 (47·5%)	1·50 (7·17 − 0·31)	0·71
	No	8	3 (37·5%)		
ADA level > 31.5 IU/ml	Yes	41	20 (48·7%)	2.38 (13.70 − 0.41)	0.42
	No	7	2 (28·5%)		

## Discussion

Diagnosis of pleural TB is mostly dependent upon combination of clinical, radiological, biochemical, and histopathological evidence in the absence of highly dependable microbiological assay. The most common methods of microbiological diagnosis, such as culture and NAAT, are often inconclusive because of their low sensitivity in detecting this paucibacillary disease. Liquid culture has been reported to have a sensitivity as low as 20% in pleural tuberculosis when compared to histopathological standards, despite its 100% specificity [12]. Whereas, CBNAAT has pooled sensitivity and specificity of 51·4% and 98·6% when compared to mycobacterial culture [13].

Lipoarabinomannan is a widely researched biomarker candidate for non-sputum-based POCT. It’s performance as a POCT on pleural fluid in the present study was inferior to the WHO target range for non-sputum based POCT [3].It showed sub-optimal sensitivity of 61.54% for diagnosis of pleural TB against MRS which further reduced to 45.83% when compared against CRS. Hence, the test was not very useful to diagnose the diseased population as a POCT. However, it definitely outperformed diagnostic ability of the currently used microbiological assays like liquid culture and NAAT with sensitivity of 66·42% and 77·78% respectively. At the same time, although less than the expected WHO target range, but the assay showed reasonably good specificity 91.67% and 95.08% against MRS and CRS respectively implying that negative result on LAM testing can reliably rule out the disease. The specificity against the individual microbiological assays was also greater than 90%. Only two authors, Dheda et al. (2009) [7] and Wang Fang et al. (2012) [8] have examined LAM performance in pleural fluid previously. Both studies used Clearview TB ELISA and showed extremely low sensitivity of 8% and 54% and good specificity of 100% and 78% in definite TB patients. LAM detection by few authors in TB meningitis [5, 6] and TB pericarditis [4], was also tested using LFA and ELISA with similar results.

The sub-optimal sensitivity of this assay can be attributed to obvious reasons like less bacillary load in the effusion as is the case for any other diagnostic modality, relatively low limit of detection (LOD) of the LAM assay in current format and due to matrix phenomenon [14]. Matrix phenomenon involves masking of the antigen due to high protein content of sterile body fluids binding with the free LAM and rendering it undetectable [15]. The perchloric acid treatment method applied to remove matrix phenomenon did not yield any results, rather in few cases the visibility of control band in LF-LAM assay was diminished as monoclonal antibodies used in lateral flow assay might have been damaged by the residual chemicals. Other pretreatment methods suitable for both the sample and methodology needs to be explored to alleviate this effect. Less than expected specificity of LAM was because of false positive results in six non -TB cases, three of which had fungal etiology of their disease. False positivity due to this type of cross interaction with a variety of bacteria, including Actinobacteria, Rhodococcus and Candida had been reported by Iskandar et al. [16].

In comparison to pleural fluid, the diagnostic accuracy of urinary LAM antigen detection was poor. It missed 21 DTB cases, including two HIV positive patients, resulting in much lower sensitivity, while picking up two false positives, lowering specificity. Less urinary excretion of the antigen due to LAM accumulation at the site of primary disease, i.e., Pleura, as well as its sequestration within extracellular vesicles (EVs) when secreted in the urine, rendering it undetectable by commercial immunoassays, may be important factors contributing for lower sensitivity [17]. Among the two false positive results, one had a proven fungal etiology, while the other had pulmonary malignancy, which increases the likelihood of opportunistic fungal infections, resulting in fungal urinary antigen secretion. Cross-reacting bacteria or fungi can also cause false positives in urine samples [18].

The intertest agreement between pleural fluid LAM and urinary LAM was fair (cohen’s kappa − 0·265). Five of the six patients who tested positive for both had a higher grade of positivity in pleural LAM. They were also discovered to have severe illnesses, with two of them being HIV positive. These findings suggest that detecting urinary LAM in patients with pleural TB is of limited utility, except in patients with high bacillary loads at the primary site or with disseminated disease. Minion et al. found 3–53% higher sensitivity in HIV-positive subgroups than HIV-negative subgroups in four studies stratified by HIV status [19]. Since we had few HIV-positive patients, we did not stratify our data by HIV status, but the current study showed a 28% increase in LAM detection from HIV-positive urine samples.

Pleural LAM detection had higher diagnostic value in malnourished patients and patients with a greater severity of disease as demonstrated by significant associations between these risk factors and pleural LAM positivity. Other associated risk factors like diabetes mellitus, concomitant pulmonary disease, duration of illness > 1 week also showed higher odds of pleural LAM positivity however, they were not statistically significant.

The significance of ADA and LDH as diagnostic biomarkers for pleural tuberculosis is well established [20, 21]. In our study, the significant AUC-derived cut point for ADA to diagnose definite TB and probable plus definite TB was determined to be 31·5 IU/L and 40·0 IU/L, respectively, which is consistent with the value reported in the literature by various studies [21]. Since ADA is a crucial adjunct to microbiological methods for diagnosing pleural TB, we investigated the performance of the LAM assay in a subset of the study population with elevated ADA levels. The most encouraging finding of this study was that the performance of the LAM assay in this subset was significantly enhanced. The sensitivity of LAM increased marginally over the original against both comparators, while its specificity against CRS reached 100%. In addition, LAM testing could accurately predict the presence of disease (PPV − 100%) in patients with pleural TB suspicion. This demonstrates that LAM has a higher diagnostic value when performed on patients with ADA levels > 40 IU/L. This can aid clinicians in making decisions for patients with a suspected disease or inconclusive or unavailable microbiological results.

The study has several limitations, including a smaller sample size due to patient loss during the COVID-19 pandemic, the non-inclusion of pleural biopsy as a standard specimen to diagnose pleural TB due to its complications and invasiveness, non-uniformity in NAAT testing due to CBNAAT procurement issues across the country, the lack of sample pretreatment to reduce matrix effect, and the use of an assay format with a lower LOD.

To the best of our knowledge, the aforementioned finding is the first of its kind to be reported in scientific literature anywhere in the world, and the study is the first from an Indian context. We recommend more research on the use of LAM antigen testing in pleural fluid of suspected pleural TB patients with ADA levels greater than 40 IU/L and utilizing newer methods/technology with better LOD and newer pre-treatment methods. Moreover, when interpreting LAM results, the possibility of false-positive results due to cross-reactivity with other LAM-like compounds must be considered.

## Conclusion

Pleural LAM antigen detection by lateral flow assay showed reasonably good diagnostic accuracy against the individual as well as combined microbiological and composite reference standards, although this was suboptimal compared to WHO recommendations for non-sputum based POCTs.

Moreover, it was found to be more useful in predicting absence of the disease if the test is negative rather than using as a POCT for diagnosis. To increase the sensitivity, it is recommended to conduct additional studies using testing formats with better LOD and newer methods of sample pre-treatment.

## Data Availability

The datasets used and/or analysed during the current study are available from the corresponding author on reasonable request.

## Abbreviations

TB: Tuberculosis
LAM: *Lipoarabinomannan*
Mtb: *Mycobacterium tuberculosis*
HIV: Human Immunodeficiency Virus
ADA: Adenosine Deaminase
MRS: Microbiological Reference Standards
CRS: Composite Reference Standards
NAAT: Nucleic Acid Amplification Test
CBNAAT: Cartridge Based Nucleic Acid Amplification Test
WHO: World Health Organization
PTB: Probable Tuberculosis
DTB: Definite Tuberculosis
NTB: Non-Tuberculosis
MGIT: Mycobacterium Growth Indicator Tube
LOD: Limit of detection
LFA: Lateral Flow assay
MBS: Modified Bandim Scoring
PPV: Positive Predictive Value
NPV: Negative Predictive Value
